# Epithelial-to-mesenchymal transition leads to disease-stage differences in circulating tumor cell detection and metastasis in pre-clinical models of prostate cancer

**DOI:** 10.18632/oncotarget.12682

**Published:** 2016-10-15

**Authors:** Lori E. Lowes, David Goodale, Ying Xia, Carl Postenka, Matthew M. Piaseczny, Freeman Paczkowski, Alison L. Allan

**Affiliations:** ^1^ Department of Anatomy & Cell Biology, Schulich School of Medicine and Dentistry, Western University, London ON, Canada; ^2^ Department of Oncology, Schulich School of Medicine and Dentistry, Western University, London ON, Canada; ^3^ London Regional Cancer Program, London Health Sciences Centre, London ON, Canada; ^4^ Lawson Health Research Institute, London ON, Canada

**Keywords:** prostate cancer, circulating tumor cells, epithelial-to-mesenchymal transition, metastasis, pre-clinical models

## Abstract

Metastasis is the cause of most prostate cancer (PCa) deaths and has been associated with circulating tumor cells (CTCs). The presence of ≥5 CTCs/7.5mL of blood is a poor prognosis indicator in metastatic PCa when assessed by the CellSearch^®^ system, the “gold standard” clinical platform. However, ~35% of metastatic PCa patients assessed by CellSearch^®^ have undetectable CTCs. We hypothesize that this is due to epithelial-to-mesenchymal transition (EMT) and subsequent loss of necessary CTC detection markers, with important implications for PCa metastasis. Two pre-clinical assays were developed to assess human CTCs in xenograft models; one comparable to CellSearch^®^ (EpCAM-based) and one detecting CTCs semi-independent of EMT status via combined staining with EpCAM/HLA (human leukocyte antigen). *In vivo* differences in CTC generation, kinetics, metastasis and EMT status were determined using 4 PCa models with progressive epithelial (LNCaP, LNCaP-C42B) to mesenchymal (PC-3, PC-3M) phenotypes. Assay validation demonstrated that the CellSearch^®^-based assay failed to detect a significant number (~40-50%) of mesenchymal CTCs. *In vivo*, PCa with an increasingly mesenchymal phenotype shed greater numbers of CTCs more quickly and with greater metastatic capacity than PCa with an epithelial phenotype. Notably, the CellSearch^®^-based assay captured the majority of CTCs shed during early-stage disease *in vivo*, and only after establishment of metastases were a significant number of undetectable CTCs present. This study provides important insight into the influence of EMT on CTC generation and subsequent metastasis, and highlights that novel technologies aimed at capturing mesenchymal CTCs may only be useful in the setting of advanced metastatic disease.

## INTRODUCTION

Prostate cancer (PCa) is the most commonly diagnosed cancer and second most common cause of cancer death in American men [1]. The majority of prostate cancer deaths result from metastasis, since current therapies are non-curative in the metastatic setting [2]. Detection of circulating tumor cells (CTCs) in the blood is correlated with metastatic disease burden and reduced overall survival [3–6], with ≥5 CTCs/7.5ml of blood being indicative of poor prognosis in metastatic PCa patients [6]. Additionally, changes in CTC number throughout treatment have been demonstrated to reflect therapy response [7].

Due to the rare nature of CTCs (~1 CTC/10^5^-10^7^ leukocytes in metastatic patients), extremely sensitive technologies are required for accurate CTC detection [8]. Several techniques have been employed to enrich CTCs including size- and/or density-based separation, and antibody-based techniques with/without the aid of microfluidics, while detection techniques rely almost exclusively on protein- (immunofluorescence/flow cytometry) or nucleic acid-based (RT-PCR/qRT-PCR) assays [9, 10]. Each approach has unique advantages and disadvantages; however most lack the standardization and quality control required for routine clinical use. The CellSearch^®^ system (CSS; Janssen Diagnostics) provides a standardized method for sensitive detection and quantification of CTCs in human blood. It is the only CTC assay approved by the U.S. Food and Drug Administration for clinical management of metastatic prostate, breast, and colon cancer patients [4–6], and is thus considered the “gold standard” CTC platform.

The CSS distinguishes CTCs from leukocytes through immunomagnetic selection of cells with an EpCAM^+^ (epithelial cell adhesion molecule) phenotype followed by differential fluorescent staining for cytokeratins (CK) 8/18/19, CD45 (leukocyte marker), and DNA (4′,6-diamidino-2-phenylindole [DAPI]). Although the CSS has been used to effectively enumerate CTCs in the blood of metastatic PCa patients [6], CTCs are undetectable in ~30% of these patients despite the presence of systemic disease [11]. This suggests that either CTCs are truly not present in ~1/3 of metastatic PCa patients; or that CTCs are present but undetectable by the CSS because they do not meet the standard CTC definition (EpCAM^+^/CK^+^/DAPI^+^/CD45^−^). It has been proposed that this lack of detection may be due to the epithelial-to-mesenchymal transition (EMT) [12–14], a dynamic cellular process that leads to downregulation of epithelial markers necessary for CTC capture/enumeration, including EpCAM/CK [12, 15, 16]. Corresponding increases in mesenchymal markers (N-cadherin/vimentin/fibronectin) and resulting changes in cellular morphology have been shown to enhance cancer invasion, metastasis, therapy resistance, and disease aggressiveness [12, 17]. The standard CSS definition of CTCs may therefore be missing the most invasive and highly metastatic cells driving disease progression. In support of this, several studies have demonstrated that CTCs with a purely mesenchymal phenotype are undetectable by the CSS, but that the presence of mesenchymal marker expression on CTCs with a hybrid epithelial-mesenchymal (E-M) phenotype is indicative of poor prognosis [15, 18–21]. This suggests that current clinical CTC assays may be limiting our ability to capitalize on the full potential of CTCs, and that a greater understanding of CTC biology is necessary in order to guide future technology development and translation to the clinic.

The field of CTC research is quite unique in that it has evolved using a “bedside-to-bench” path rather than the more traditional “bench-to-bedside” model of translational research. Although this has allowed for quick entry of CTC technology into the clinic, outstanding questions regarding the biology of CTCs has resulted in a hesitance for their widespread adoption as biomarkers for directing patient care. Unfortunately, appropriate experimental tools needed to address these outstanding biology questions have been largely lacking, especially those that mimic the approach utilized by the clinically-used CSS. This highlights the need for implementation of suitable pre-clinical models and development of complementary CTC analysis techniques that can assess not only the CTCs which are captured using the CSS but also those that may be missed, in order to advance knowledge. Previous work investigating CTC biology in our laboratory using orthotopic xenograft models demonstrated that CTC dissemination occurs relatively early in the metastatic cascade and that CTCs can be generated by both primary tumors as well as metastases [22–24]. However, very little is currently known about the functional role of EMT in CTC generation, detection and metastasis, particularly in the context of prostate cancer.

In this study, we hypothesized that the EpCAM-based CSS assay is unable to detect CTCs that have undergone EMT, and that EMT-related phenotypic differences in CTCs have important implications for PCa disease progression. To test this, we developed two pre-clinical assays for assessing human CTCs in xenograft models; one that is comparable to the EpCAM-based CSS (dependent on EMT status) and one that detects CTCs semi-independent of EMT status via negative depletion of CD45 and combined staining with EpCAM and HLA (human leukocyte antigen). Using these assays, differential *in vivo* CTC generation, capture efficiency, kinetics and metastasis were assessed using 4 human PCa cell lines (LNCaP, LNCaP C4-2B, PC-3, PC-3M) of increasing aggressiveness in pre-clinical orthotopic models of PCa. The novel results presented here provide functional evidence of the interplay between EMT and CTC biology, shedding light on which CTCs are the most important to study. This knowledge has the potential to inform ongoing CTC technology development and guide strategies for the most effective use of CTCs as prognostic/predictive biomarkers in clinical oncology.

## RESULTS

### Human PCa cell lines display differences in EMT phenotype

**Figure 1:**
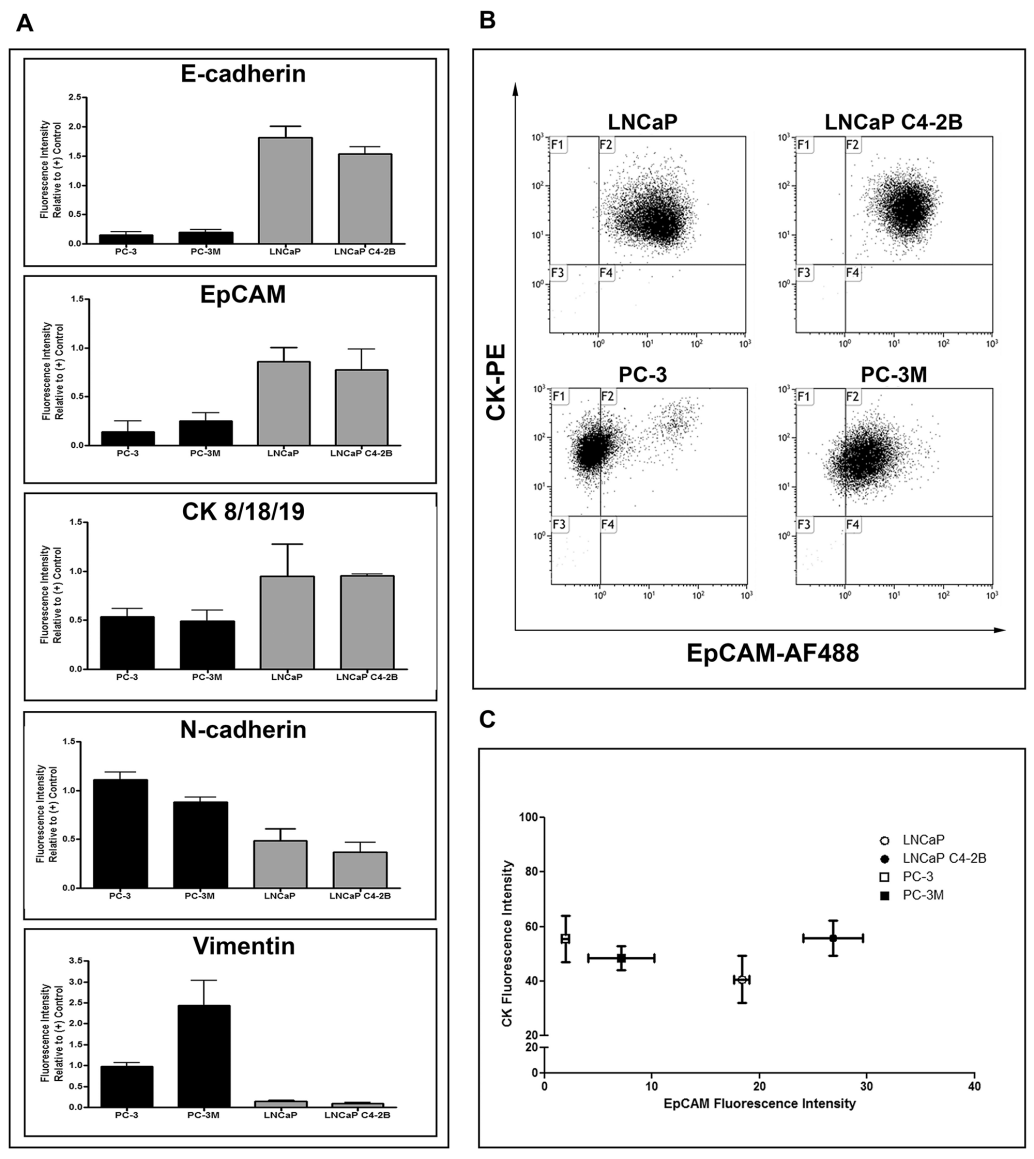
Human prostate cancer cell lines display differences in EMT phenotype **A.** Protein expression analysis by flow cytometry for the epithelial-associated markers E-cadherin and EpCAM and the mesenchymal-associated markers N-cadherin and vimentin in PC-3M, PC-3, LNCaP C4-2B, and LNCaP human prostate cancer cells. Data are presented as relative fluorescence intensity (expression) compared to appropriate positive control cell lines (E-cadherin/EpCAM/CK: MDA-MB-468; N-cadherin/vimentin: HeLa) (n=3). The expression of epithelial-associated and mesenchymal-associated proteins corresponds to previously reported cell aggressiveness and *in vivo* metastatic capacity of these cell lines. **B.** Flow cytometry dot plots of the differential expression of EpCAM (AF488) and CK8/18/19 (PE) in investigated prostate cancer cell lines. **C.** Flow cytometry analysis for co-expression of EpCAM and CK8/18/19, presented as the mean ± SEM fluorescence intensity of the investigated proteins for each cell line (n=3).

Four human PCa cell lines (LNCaP, LNCaP C4-2B [C4-2B], PC-3, PC-3M) previously reported to have progressively increasing metastatic capacity [25–28] were characterized for epithelial (E-cadherin/EpCAM/CK) and mesenchymal (N-cadherin/vimentin) markers using qRT-PCR, immunoblotting (Supplementary Figure S1A, 1B), and flow cytometry (FCM) (Figure 1A). LNCaP and C4-2B had consistently higher protein expression of epithelial-associated markers E-cadherin and CK8/18/19, while PC-3 and PC-3M had consistently higher protein expression of mesenchymal-associated markers N-cadherin and vimentin. Although EpCAM levels appeared similar between cell lines at the mRNA level (Supplementary Figure S1A), differences in EpCAM protein expression were evident, with LNCaP and C4-2B demonstrating higher levels compared to PC-3 and PC-3M (Supplementary Figure S1B, Figure 1A). To further investigate potential capacity for capture of these cells by the EpCAM- and CK-reliant CSS, protein co-expression was assessed using FCM (Figure 1B, 1C). This further confirmed differential EpCAM expression between cell lines, but interestingly demonstrated a similar distribution of CK8/18/19 expression, suggesting that any differences in CTC capture between cell lines would be due to differences in EpCAM expression rather than CK8/18/19.

The ability of E-cadherin to maintain the epithelial phenotype and normal adhesive function of cells is dependent on its localization to the cell membrane [29, 30]. We observed that that although E-cadherin was expressed in PC-3, it was aberrantly localized to the cytoplasm, likely due to a lack of α-catenin expression which is necessary for appropriate E-cadherin membrane localization [31]. In contrast, LNCaP and C4-2B strongly expressed E-cadherin with appropriate membrane localization (Supplementary Figure S2).

### CTC recovery using the CSS is significantly reduced for PCa cells with a mesenchymal phenotype

As the current gold standard CTC detection technology in the clinical setting, the CSS relies solely on the epithelial-associated marker EpCAM for CTC capture. However, EpCAM has been shown to be downregulated in cells with an invasive phenotype [32], suggesting that EpCAM-based CTC detection techniques such as the CSS may be missing a portion of the CTCs that enter the bloodstream. To assess this, we developed 2 novel pre-clinical CTC assays for use with xenograft models; one that recapitulates EpCAM-based capture of CTCs by the CSS (“EMT-dependent”), which captured CTCs based on an EpCAM+/CK+/CD45-/HLA+ phenotype, and one designed to detect all the CTCs shed into the circulation regardless of EMT status (“EMT semi-independent”), capturing CTCs based on a joint human HLA/EpCAM approach, including EpCAM^low/−^ cells (PC-3, PC-3M; likely captured primarily by HLA) and EpCAM^+^ but HLA^variable/low^ cells (LNCaP, C4-2B; likely captured primarily by EpCAM). Use of the EMT-dependent assay resulted in significantly reduced recovery of CTCs with mesenchymal phenotypes (PC-3/PC-3M) when compared to CTCs with epithelial phenotypes (LNCaP/C4-2B) (p≤0.05) (Supplementary Figure S3A). However, when the EMT semi-independent assay was utilized, although overall CTC recovery was lower compared to the EMT-dependent assay, percent recovery was not significantly different across cell lines regardless of EMT status (Supplementary Figure S3B). The reduced recovery demonstrated by the EMT semi-independent assay was further investigated by incorporating the additional sample handling steps required for the EMT semi-independent assay (i.e. red blood cell lysis and additional washes) into the EMT-dependent assay. The results demonstrated that when using the same reagents and highly epithelial cells (C4-2B), addition of extra processing steps resulted in equivalent sample loss between matched samples when comparing both assays (Supplementary Figure S3C).

### Prostate cancer cell lines with an increasingly mesenchymal phenotype have an enhanced capacity for CTC shedding *in vivo* and produce CTCs that are undetectable by the CSS

**Figure 2:**
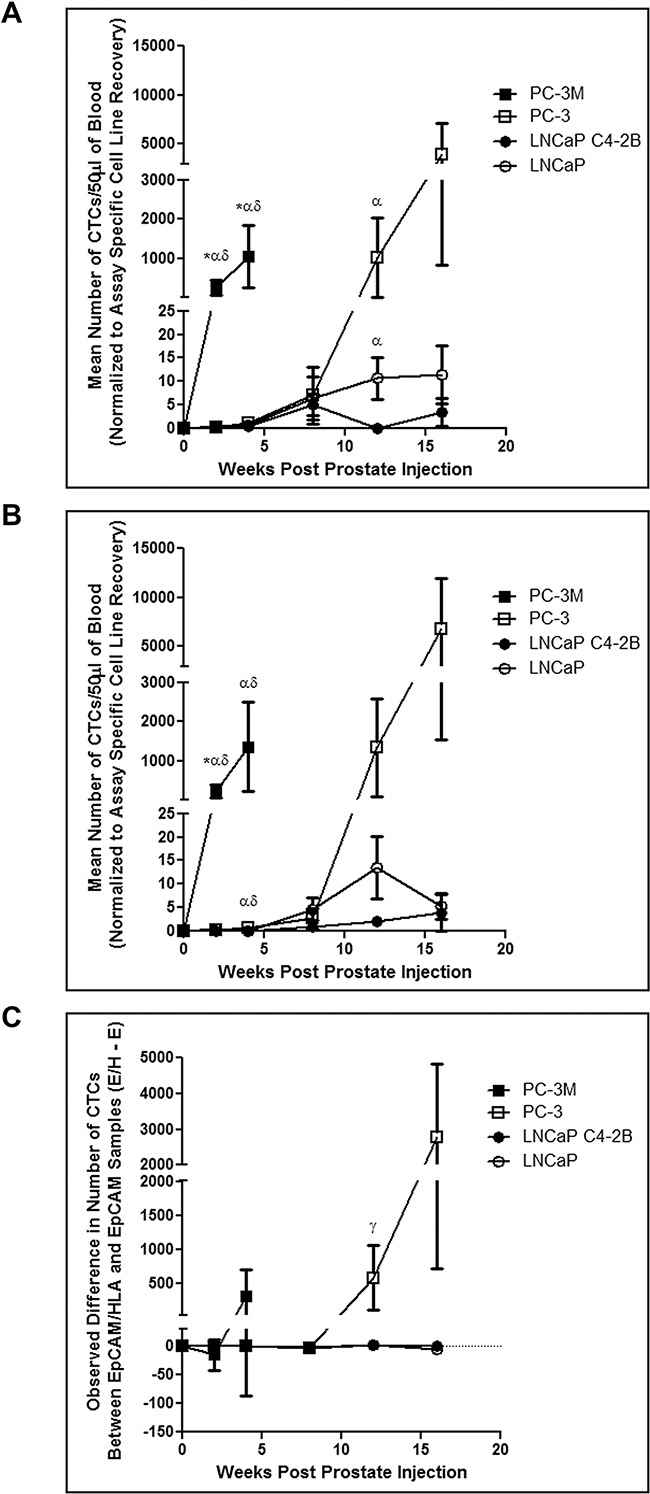
Human prostate cancer cell lines with an increasingly mesenchymal phenotype shed greater numbers of CTCs more quickly and have an enhanced *in vivo* capacity for shedding CTCs that are undetectable by the CellSearch^®^ system PC-3M, PC-3, LNCaP C4-2B, and LNCaP human prostate cancer cells were orthotopically injected into 6-8 week old male nude mice via the right dorsolateral lobe of the prostate (1×10^6^ cells/mouse) to assess spontaneous metastasis. At several timepoints post injection (2, 4, 8, 12, and 16 weeks) mice were sacrificed and blood (100μl) was collected and processed using both the **A.** EMT-dependent and **B.** EMT semi-independent assays (50μl/assay) to assess differences in CTC recovery. Data are presented as the mean ± SEM (n=5-12 mice/group). **C.** Comparison of the observed difference in the number of CTCs detected using the EMT-dependent and EMT semi-independent assays in matched samples. Data are presented as the mean (± SEM) difference in the number of observed CTCs between both assays (# captured by EpCAM/HLA assay - # captured by EpCAM assay) at a given timepoint (n=5-12 mice/group). Positive values represent groups in which more CTCs were detected with the EMT semi-independent assay, whereas negative values represent groups in which more CTCs were detected with the EMT-dependent assay. Differences in the mean number of CTCs between cell lines at a given timepoint using and differences between each assay within individual mice was assessed using Wilcoxon Scores followed by a Kruskal-Wallis test at each timepoint. Comparison of differences between each assay in matched data sets within cell lines at a given timepoint was performed using a Wilcoxon matched-pairs signed rank test. * = significant difference relative to PC-3; α = significant difference relative to LNCaP C4-2B; δ = significant difference relative to LNCaP; γ = overall significant difference (unable to perform pairwise comparison) (p≤0.05).

LNCaP, C4-2B, PC-3, and PC-3M cells prepared in Hank's buffered saline were injected (1×10^6^ cells/40μL per mouse) orthotopically via the right dorsolateral lobe of the prostate as described in the Materials and Methods in order to assess *in vivo* CTC generation, kinetics, and detection by EMT-dependent versus EMT semi-independent assays, and relationship to subsequent metastasis. Using both CTC assays, we observed that highly mesenchymal PC-3M shed CTCs very quickly post-injection and in greater numbers than all other cell lines at most timepoints (p≤0.05) (Figure 2A). Additionally, mesenchymal-like PC-3 shed a similar number of CTCs as epithelial LNCaP and C4-2B until week 4 (EMT semi-independent assay) or week 12 (EMT-dependent assay), at which time the number of CTCs increased significantly (p≤0.05). To quantify differences in CTC recovery based on EMT status, normalized CTC values obtained using both assays from each timepoint were generated, and numbers of CTCs identified using the EMT-dependent assay were subtracted from numbers of CTCs identified using the EMT semi-independent assay and presented as the mean for each timepoint. Positive values represent instances where more CTCs were detected with the EMT semi-independent assay, whereas negative values represent instances where more CTCs were detected with the EMT-dependent assay (Figure 2C). We observed that epithelial LNCaP and C4-2B had similar CTC recovery across both assays at all timepoints investigated. However, mesenchymal PC-3 and PC-3M showed increased numbers of CTCs recovered using the EMT semi-independent assay at later timepoints, with significant differences observed when comparing PC-3 to LNCaP and C4-2B at 12 weeks (p≤0.05).

### Prostate cancer cell lines with an increasingly mesenchymal phenotype have enhanced capacity for primary tumor formation and metastasis

**Figure 3:**
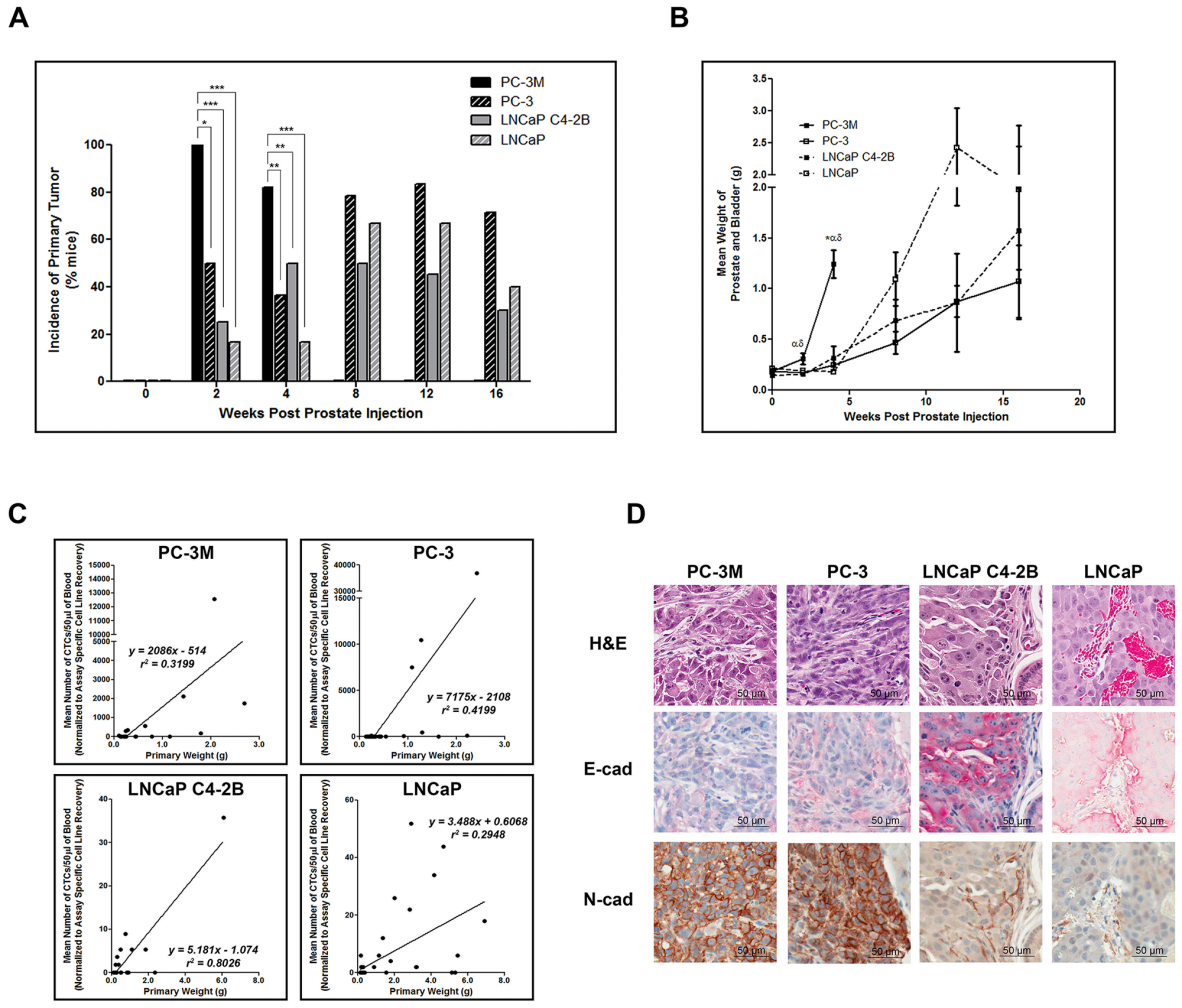
Highly mesenchymal prostate cancer cells exhibit enhanced primary tumor incidence and size and in all cell lines CTC number is correlated with primary tumor size **A.** Incidence of primary tumor formation following orthotopic injection of PC-3M, PC-3, LNCaP C4-2B, and LNCaP human prostate cancer cell lines. Data are presented as the percentage of mice per cell line per timepoint with detectable primary tumors (n=6-39 mice/group) as assessed by microscopic histological examination of formalin-fixed, H&E stained tissue. * = significantly different (p<0.05), ** (p<0.01), *** (p<0.001). **B.** Mean combined weight of prostate and bladder at time of sacrifice following orthotopic injection of prostate cancer cell lines. Data are presented as the mean ± SEM (n=6-39 mice/group). **C.** Mean normalized number of CTCs/50μl of blood (assessed using the EMT semi-independent assay) correlates with the primary tumor weight in all cell lines. **D.** Representative H&E and IHC (E-cadherin and N-cadherin) images of primary prostate tumors for each cell line (40x; scale bars = 50 μm). Primary tumor weights were assessed for cell line variances using Levene's test, followed by a 1-way ANOVA with Tukey's post-test for multiple comparisons. Spearman rank correlation was utilized to examine the relationship between mean number of CTCs and primary tumor weight. * = significant difference relative to PC-3; α = significant difference relative to LNCaP C4-2B; δ = significant difference relative to LNCaP (p≤0.05).

To determine differences in the extent of disease and metastatic spread across the 4 cells lines, at necropsy, animals were assessed for primary tumor incidence/weight, and metastatic spread to lymph nodes and distant metastatic sites (lung, liver, bone). We observed that primary tumor incidence and size was significantly increased in highly mesenchymal PC-3M compared to all other cell lines (p≤0.05), except when considering tumor weight of PC-3 at 2 weeks (Figure 3A, 3B). All other cell lines showed comparable primary tumor incidence and weight at all timepoints. Additionally, primary tumor weight and the number of CTCs shed into the circulation (EMT-semi-independent assay) were positively correlated for all cell lines (Figure 3C). Immunohistochemical analysis of E-cadherin/N-cadherin revealed that all cell lines demonstrated comparable epithelial/mesenchymal phenotypes *in vivo* relative to those seen *in vitro* (Figure 3D, Supplementary Figure S4A).

Additional differences between mesenchymal and epithelial cell lines were observed in metastatic incidence (% of mice developing metastasis) and metastatic burden (% of organ occupied by metastatic tumor) to the lymph nodes (LN). Microscopy analysis revealed that PC-3M had significantly increased incidence of LN metastases versus all other cell lines at 4 weeks (p≤0.05), while PC-3 had significantly increased incidence at 8 and 12 weeks compared to C4-2B (p≤0.05). Interestingly, the incidence of metastases to LN did not differ significantly between PC-3 and LNCaP (Supplementary Figure S5A). However, PC-3 did demonstrate significantly increased metastatic burden compared to C4-2B at 8 and 12 weeks (p≤0.05) (Supplementary Figure S5B). Therefore, although these cell lines appear to have a similar capacity to disseminate to LN, they do not have the same capacity for subsequent growth in this organ. Finally, LNCaP demonstrated significantly increased metastatic burden compared to C4-2B at 12 weeks (p≤0.05). Representative H&E and IHC analysis of LN metastases are shown in Supplementary Figure S4B, S5C.

**Figure 4:**
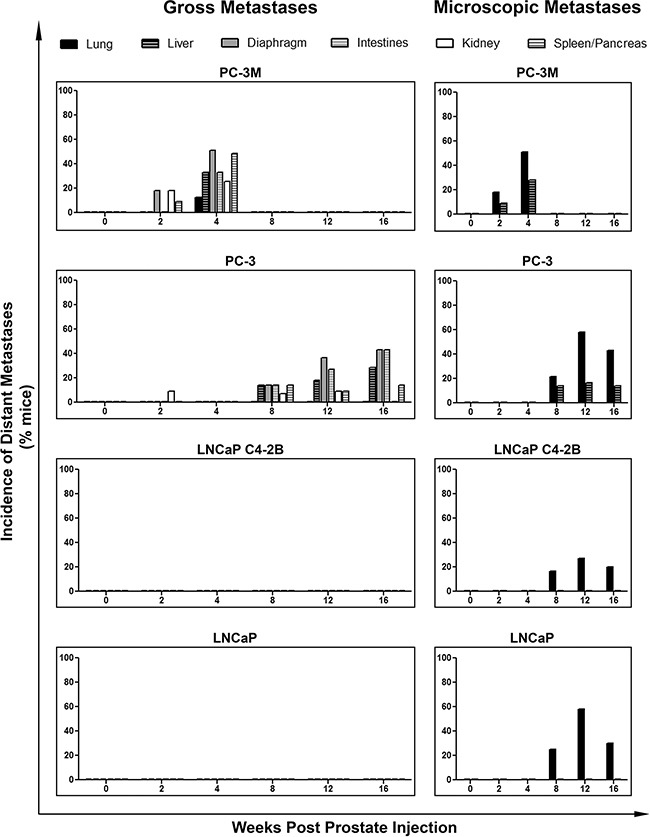
Human prostate cancer cell lines with an increasingly mesenchymal phenotype have an enhanced *in vivo* capacity for metastasis to distant organs Incidence of metastasis to distant organs following orthotopic injection of PC-3M, PC-3, LNCaP C4-2B, and LNCaP human prostate cancer cell lines. Data are presented as the percentage of mice per cell line per timepoint with detectable distant metastases to the lung, liver diaphragm, intestines, kidney, and/or spleen/pancreas (n=7-39 mice/group) as assessed by gross observation at necropsy (*left panel*) and microscopic histological examination (*right panel*) of tissues.

**Figure 5:**
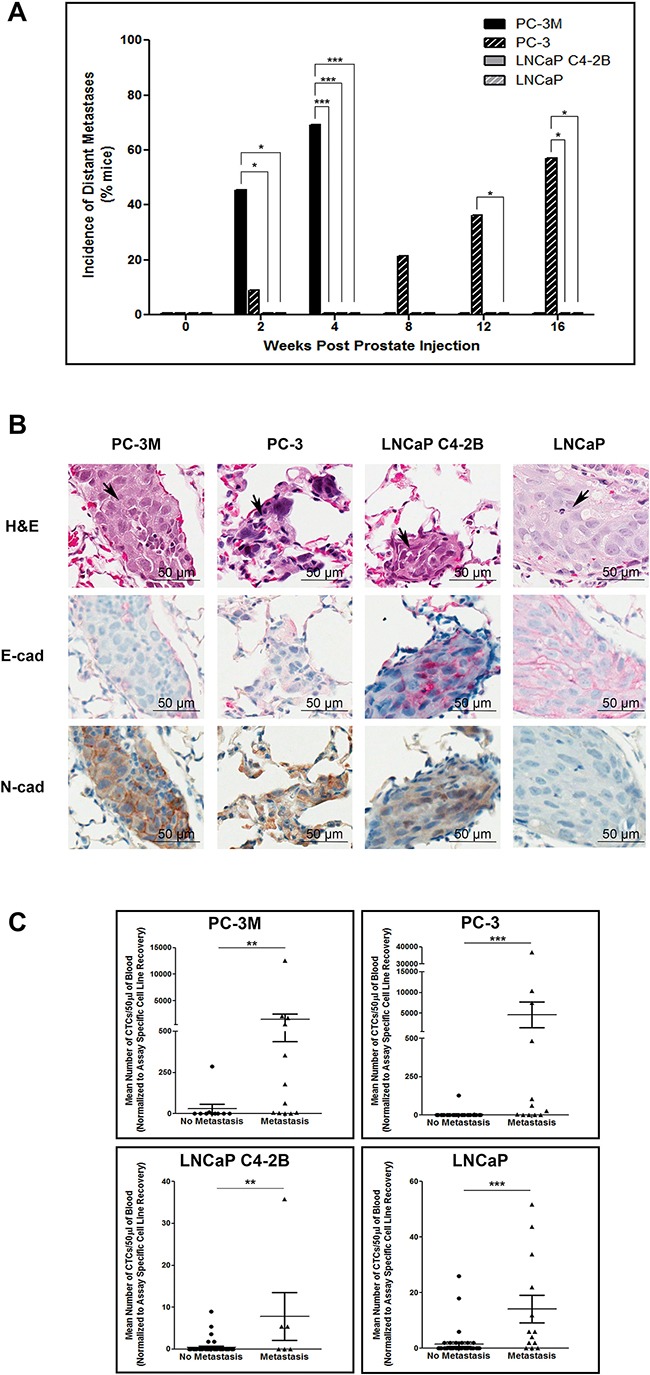
Mesenchymal human prostate cancer cell lines exhibit an enhanced capacity for metastasis that is correlated with CTC dissemination **A.** Incidence of metastasis to distant organs following orthotopic injection of PC-3M, PC-3, LNCaP C4-2B, and LNCaP prostate cancer cell lines. Data are presented as the percentage of mice per cell line per timepoint with detectable distant metastases (n=7-39 mice/group) as assessed by gross observation at necropsy. **B.** Representative H&E and IHC (E-cadherin and N-cadherin) images of lung metastases for each investigated cell line. Histological sections are presented at 40x magnification. Arrowheads on H&E images indicate regions of tumor within the given tissue. Scale bars = 50 μm. **C.** Correlation of CTC number and incidence of metastasis. Mean number of CTCs/50μl of blood, assessed using the EMT semi-independent assay, are presented for mice with either metastasis to the lymph nodes or any distant organ (based on gross and/or microscopic analysis) or mice with no evidence of metastasis at any timepoint (n=6-47 mice/group). Differences in the incidence of primary tumors, lymph node metastasis, and distant metastasis were assessed using Fisher's Exact Test. Differences in the mean number of CTCs in mice with no metastasis versus those with metastatic disease were compared using a Student's t-test. * = significantly different (p≤0.05), ** (p<0.01), *** (p<0.001).

Differences in distant metastasis were investigated by gross assessment at necropsy and microscopic assessment following H&E staining. We observed that both PC-3M and PC-3 could disseminate to and establish gross macrometastases in a number of distant organs, while no visible macrometastases were observed at necropsy in mice injected with either LNCaP or C4-2B (Figure 4). However, microscopy analysis of lung and liver revealed distant metastases to these organs for all of the investigated cell lines. Analysis of the incidence of gross metastatic disease demonstrated that more mesenchymal PC-3M and PC-3 had significantly increased metastatic capacity versus more epithelial LNCaP and C4-2B (p≤0.05) (Figure 5A). Representative H&E and IHC analysis of lung metastases are shown in Figure 5B and Supplementary Figure S4C. Additionally, the number of CTCs was significantly higher across all cell lines in mice with metastatic disease relative to those without (p≤0.05) (Figure 5C), and in those cell lines with the greatest metastatic capacity, thus demonstrating the relationship between CTCs and metastatic spread.

### Circulating tumor cells acquire a more mesenchymal phenotype during disease progression

**Figure 6:**
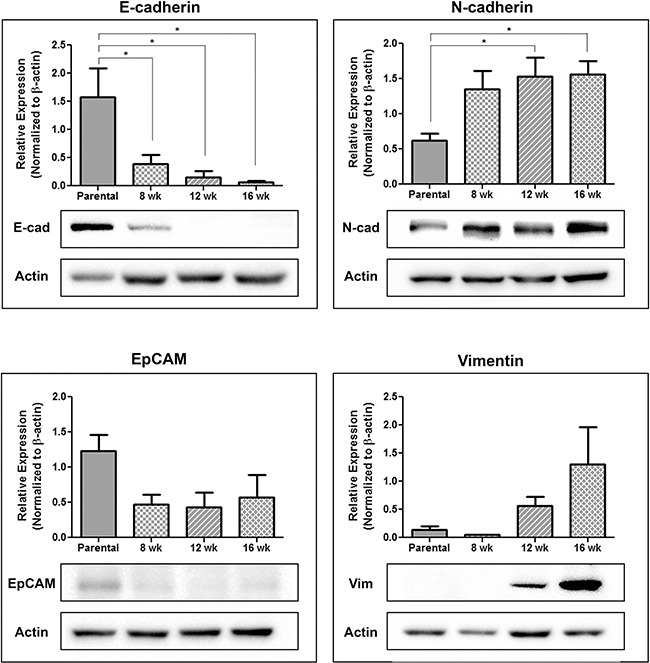
Circulating tumor cells acquire a more mesenchymal phenotype during disease progression Following orthotopic injection of PC-3 prostate cancer cells into the right dorsolateral lobe of the prostate (1 × 10^6^ cells/mouse) blood collected at 8 weeks, 12 weeks, and 16 weeks post-injection was lysed with sterile 1x NH_4_Cl, washed with PBS, and plated for tissue culture. Following 1-2 weeks of growth, with regular media changes to remove contaminating blood cells, the remaining CTCs were assessed using immunoblot for the expression of the epithelial-associated markers E-cadherin and EpCAM and the mesenchymal-associated markers N-cadherin and vimentin. Results are presented in quantitative densitometric form normalized to β-actin and as representative immunoblots, shown as cropped gel images (n=3). Changes in EMT gene expression were compared to the parental cell line using 1-way ANOVA with Dunnett's post-test for multiple comparisons. * = significantly different than parental line (p≤0.05).

To further investigate the EMT profile of CTCs shed into the circulation, blood was used to generate CTC sub-lines representing different timepoints along the metastatic cascade. Briefly, excess blood, not required for CTC assessment, was lysed (NH_4_Cl), washed, and resuspended in cell line specific culture medium. Cells were cultured, with frequent media changes to remove contaminating white blood cells, and subsequently assessed for EMT marker expression using immunoblotting. Unfortunately due to low numbers of CTCs collected from LNCaP and C4-2B, CTC growth following plating did not occur. However several sub-lines were created for both PC-3 and PC-3M. Immunoblot analysis (Figure 6) demonstrated a significant reduction in E-cadherin expression in CTCs collected at all timepoints compared to the parental PC-3 (p≤0.05), a trend in reduction of EpCAM expression, a significant increase in N-cadherin expression at later timepoints (p≤0.05), and a trend towards increasing vimentin expression, suggesting CTCs may become more mesenchymal as disease progresses.

## DISCUSSION

CTCs have emerged as a promising biomarker for tracking disease progression and therapy response in patients with different types of solid tumors. However, although CTCs are now used clinically for prognostication in metastatic prostate, breast and colorectal cancer, their underlying biology and the complex interplay between EMT, CTCs and metastasis remains poorly understood. Therefore significant debate remains with regards to which CTCs are the most valuable to capture and characterize, and which will serve as optimal tools for personalized cancer treatment [33]. The lack of knowledge about CTC biology stems from both the unique bedside-to-bench approach employed in the CTC field and the lack of appropriate tools for studying CTCs *in vivo* in pre-clinical metastasis models.

This study aimed to address these gaps through development of two novel pre-clinical CTC enumeration assays and their implementation for determining differences in CTC detection using an epithelial-based (EMT-dependent) method and a human versus mouse-based (EMT semi-independent) approach to assess the generation of mesenchymal CTCs that would be missed by current technologies, particularly the clinically used CSS. Although a previous experimental study examined CTCs in mouse models using pre-engineered, fluorescent-expressing PC-3 PCa cells [34], this is not a clinically-relevant scenario and thus we instead aimed to assess CTCs using assays modeled after those used clinically. By leveraging the capabilities of the existing CSS clinical platform and taking advantage of the HLA complex in separating human from mouse cells, the assays described here are the first report of adaptation of the CSS for use in xenograft models, without mis-identification of mouse epithelial cells. In addition, these assays allow for the processing/analysis very low volumes of blood (50μl), making these assays amenable to both blood collected at necropsy as well as serial monitoring of xenograft models in which less than 100μl aliquots of blood can be typically collected.

In the current study, assay validation using sensitivity/recovery studies demonstrated that the CSS-based assay failed to detect a significant number (~40-50%) of mesenchymal CTCs. *In vivo*, PCa tumors with an increasingly mesenchymal phenotype shed greater numbers of CTCs more quickly and with greater metastatic capacity than those with an epithelial phenotype. Notably, the CSS-based assay captured the majority of CTCs shed during early-stage disease regardless of EMT status of the originating tumor, and only after the establishment of metastases were a significant number of undetectable mesenchymal CTCs present. It is important to note that the EMT semi-independent assay, although novel and innovative, is unfortunately not adaptable for use with human blood samples. However, significant effort is being focused on the development of CTC capture techniques capable of recovering not only CTCs expressing epithelial markers (EpCAM/CK), but also (or even instead) those with a highly mesenchymal phenotype [9, 10] in human specimens. This latter pursuit is based on the idea that mesenchymal CTCs are the “bad actors”; a hypothesis supported by experimental studies demonstrating that EMT imparts enhanced invasiveness, metastatic capacity, and therapy resistance [9, 10, 12]. Efforts to detect these “bad actors” have included the exploitation of various properties of CTCs that are either independent or less reliant on their EMT status (e.g., size/deformability [microfiltration/microfluidics], electrical properties [dielectrophoresis], immunomagenetic approaches using organ/tumor specific-antigens [carcinoembyronic antigen (CEA), epidermal growth factor receptor (EGFR), prostate specific antigen (PSA), mucin-1 (MUC-1)], and adhesion assays based on CTCs ability to adhere to the presented capture surface) [35]. However, our results demonstrate that although prostate tumors with mesenchymal phenotypes shed CTCs earlier and in greater numbers than those with epithelial phenotypes, the majority these CTCs are still captured by the CSS, at least before the establishment of metastatic disease. This indicates that CTCs shed early in disease may have a hybrid EMT phenotype during dissemination, while still expressing sufficient levels of EpCAM and CK8/18/19 for detection using epithelial-based techniques. Therefore, CTCs with an E-M hybrid (rather than purely mesenchymal) phenotype may be important for establishing metastasis and therefore most interesting to characterize, at least in early-stage patients. This is supported by observations that CTCs with a hybrid phenotype may be of particular importance in the clinical setting based on their EMT/MET phenotypic plasticity [19].

Our data further demonstrates a significant increase in the number of mesenchymal CTCs that are undetectable by the CSS following the establishment of distant metastases. This increase in mesenchymal characteristics of CTCs in late-stage disease has also been demonstrated in patients with metastatic versus localized disease [36, 37]. However, further studies are needed to determine if/how these undetectable CTCs are contributing to disease progression and metastasis. This is because, despite widespread speculation, there is little evidence to support the hypothesis that highly mesenchymal CTCs have any additional prognostic/predictive value compared to hybrid E-M or even purely epithelial CTCs in patients. However, we must consider that technological limitations related to mesenchymal CTC capture may significantly hinder testing of this hypothesis in the clinic. In addition, we cannot rule out the possibility that highly mesenchymal CTCs are present in early-stage disease but not in high enough numbers to significantly contribute to differences between the 2 CTC assays described. In fact, the cancer stem cell (CSC) hypothesis posits that only a fraction of cells within the primary tumor efficiently complete the metastatic process [38]. Therefore it is possible that the dramatic increase in mesenchymal CTCs following the development of metastases is due to selective outgrowth of CSCs [39]. Thus, although this study provides valuable insights into the role of EMT in CTC dissemination/kinetics, many questions remain, for which the assays developed here will be very useful in answering.

In addition to this study's contributions towards understanding CTC biology and its relationship to EMT, to our knowledge it is the first comprehensive head-to-head comparison of EMT characteristics and *in vivo* behavior (including CTC dissemination/kinetics) of 4 commonly used PCa cell lines using orthotopic injection models of PCa. It is important to note that the metastatic process may differ in *in vivo* spontaneous metastasis models in comparison to the actual disease setting in a patient (e.g., differences in the antigen expression/tissue architecture impacting intravasation/extravasation, and decreased immune-surveillance in immune-compromised models) and therefore the selection of appropriate metastasis models which best recapitulate disease progression and dissemination is an important consideration [40–42]. The orthotopic model utilized throughout this study provides a much more clinically relevant model of CTC production and metastasis than more commonly used (and technically less challenging) subcutaneous injection models [34]. Based upon this careful selection, we anticipate that the data presented here will serve as a valuable tool for future PCa research.

Overall, our pre-clinical studies provide important translational information that will inform the use of CTCs as valuable biomarkers of cancer progression in the clinic. In particular, our data highlights that how CTC capture/characterization is utilized in the clinic may greatly depend on disease stage. Specifically, in early-stage patients where CTCs could provide tremendous value for predicting metastasis (i.e. adjuvant setting), detection of an increased number of CTCs may not require technology designed to capture mesenchymal CTCs, but instead processing of additional blood (>7.5mL) on epithelial-based CTC technologies (e.g., CSS) may suffice [43, 44]. In addition, our data supports the idea that primary tumors with an increasingly mesenchymal phenotype may have enhanced metastatic capacity and therefore the detection of CTCs with a hybrid E-M phenotype may be of prognostic/predictive importance in early-stage patients. In contrast, in late-stage disease we have demonstrated a significant increase in undetectable and highly mesenchymal CTCs after the establishment of distant metastasis. Therefore further research in this patient cohort will need to examine the functional role of these CTCs versus those with an epithelial or hybrid phenotype in disease progression and, importantly, in therapy resistance. Taken together, the results presented here provide novel and important insight into the functional influence of EMT on CTC generation and metastasis in PCa. Ultimately a better understanding of CTC biology will aid in identifying CTCs that will be most valuable for determining individualized treatment of metastatic cancer.

## MATERIALS AND METHODS

### Cell culture

LNCaP [45] (ATCC, Manassas, VA) and PC-3M [46] (a gift from Paula Foster, Western University, London, ON) human PCa cells were maintained in RPMI-1640+10% fetal bovine serum (FBS). LNCaP C4-2B [47] [C4-2B] human PCa cells (a gift from Katherine Stemke Hale, M.D. Anderson, Houston, TX) were maintained in T-media+10% FBS. PC-3 human PCa cells [48] (ATCC) were maintained in F12K media+10%FBS. MDA-MB-468 human breast cancer cells [49] (a gift from Janet Price, M.D. Anderson) were maintained in αMEM+10%FBS. HeLa human cervical cancer cells [50] (a gift from Jim Koropatnick, Western University) were maintained in DMEM+10%FBS. Media/reagents and FBS were obtained from Life Technologies (Carlsbad, CA) and Sigma (St. Louis, MO), respectively. Cell lines were authenticated via third-party testing (CellCheck, IDEXX BioResearch, Columbia, MO) in December 2015.

### Real-time PCR

RNA was isolated using TRIzol (Life Technologies), reverse-transcribed and subjected to quantitative reverse-transcription polymerase chain reactions (qRT-PCR) using Brilliant II SYBR Green qPCR Master Mix (Agilent Technologies, Santa Clara, CA) on a Stratagene Mx3000P (Life Technologies) (primer/cycling details; Supplementary Table S1). Samples were normalized using pooled qPCR human reference total RNA (Agilent Technologies) [51].

### Immunoblotting

Cells were harvested by cell scraping (E-cadherin, N-cadherin) or trypsinization (vimentin, EpCAM, α-catenin) and collected in 1% NP-40 lysis buffer. Protein (10μg) was subjected to sodium dodecyl sulfate polyacrylamide gel electrophoresis (SDS-PAGE) and transferred onto polyvinylidene difluoride membranes (PVDF; Millipore, Billerica, MA). Membranes were blocked (5% skim milk in TBS-T [Tris-buffered saline+0.1%Tween-20]; Sigma). Primary antibodies, diluted in 5% skim milk in TBS-T are described Supplementary Table S2. Goat-anti-mouse-IgG and goat-anti-rabbit-IgG secondary antibodies (Calbiochem, Billerica, MA) conjugated to horseradish peroxidase and diluted in 5% skim milk in TBS-T were used at 1:2,000 for all proteins except E-cadherin (1:10,000). Protein expression was visualized using Amersham ECL Prime Detection Reagent (GE Healthcare, Wauwatosa, WI), and normalized to total protein based on amido black (Sigma) membrane staining.

### Flow cytometry

Cells (5×10^5^) were treated with the IntraPrep™ Fix/Perm kit (Beckman Coulter, Fullerton, CA, USA) and incubated with blocking buffer (PBS/5% BSA [bovine serum albumin]); 15min). Cells were incubated with primary antibodies (30min) as detailed in Supplementary Table S3, followed by incubation with AlexaFluor488-conjugated goat-anti-mouse IgG or AlexaFluor488-conjugated goat-anti-rabbit IgG secondary antibodies (1μg; Life Technologies). Samples were analyzed using an EPICS XL-MCL or Cytomics FC500 flow cytometer (Beckman Coulter).

### Immunofluorescence

Cells were seeded into glass chamber slides (Thermo Scientific) pre-coated with fibronectin (5μg/mL; Santa Cruz Biotechnologies, Dallas, TX), grown until confluent, fixed with 2% paraformaldehyde, permeabilized (0.1% Triton X-100; Sigma) and blocked (PBS/1% BSA; 1hr) prior to incubation with anti-E-cadherin and/or anti-α-catenin primary antibodies (1:50; 1hr). Cells were labeled with goat-anti-mouse-IgG-AlexaFluor488 and/or goat-anti-rabbit-IgG-AlexaFluor594 (Life Technologies; 1:300; 1hr) before mounting with VectaShield+DAPI (Vector Laboratories, Burlingame, CA). Imaging was performed (60x) using an Olympus Provis AX70 microscope (Olympus, Richmond Hill, ON).

### Pre-clinical CTC assay development

Whole blood (100μL minimum) was collected from 6-8wk old male athymic nude (*nu/nu*) mice (Harlan Sprague-Dawley, Indianapolis, IN) via cardiac puncture as described [22, 23]. Blood was processed immediately or stored for up to 48 hours with an equal volume of CytoChex (Streck, Omaha, NE). For CTC assay development, 50μL of blood was “spiked” with 1000 tumor cells. To assess recovery of low numbers of cells (5-100), serial dilutions were performed (*data not shown*).

The EMT-dependent CTC assay was adapted from the Veridex mouse/rat CellCapture kit (no longer available) as described previously [52]. Briefly, 50μL of whole blood was incubated with components of the CellSearch^®^ CTC kit; including anti-EpCAM ferrofluid, Capture Enhancement Reagent, Nucleic Acid Dye, Staining Reagent, and Permeabilization Reagent. Additional reagents included anti-mouse-CD45-APC (0.30μg; 30-F11; eBioscience, San Diego, CA), and anti-human-HLA-AlexaFluor488 (1.5μg; W6/32; BioLegend, San Diego, CA). Samples were immune-magnetically separated and transferred to a MagNest™ for analysis using the CSS. EpCAM^+^/CK^+^/DAPI^+^/CD45^−^/HLA^+^ cells with a round/oval morphology were classified as CTCs.

Development of the EMT semi-independent CTC assay was based on negative selection/immunodepletion of CD45^+^ leukocytes combined with dual staining with human HLA and EpCAM. Our initial assay design only employed HLA (to take advantage of the human-in-mouse model), however we observed that relative levels of HLA present on each of the cell lines was highly variable, and in particular the EpCAM^+^ LNCaP and C4-2B cells had low HLA compared to PC3 cells (*data not shown*). Since differences in HLA would makes interpretation of CTC capture data very difficult, we took a joint HLA/EpCAM approach which detects all CTCs present, including EpCAM^low/−^ cells (PC-3, PC-3M; by HLA) and EpCAM^+^ but HLA^variable/low^ cells (LNCaP, C4-2B; by EpCAM). For testing, 50μL of blood was lysed with NH_4_Cl. Samples were washed and labeled (20min) using anti-human-HLA-PE (0.2μg; W6/32; BioLegend), anti-human-EpCAM-PE (0.0075μg; EBA-1; BD Bioscience), and anti-mouse-CD45-APC (0.30μg). Samples were washed and immunomagnetically enriched using the EasySep APC Positive Selection kit (StemCell Technologies, Vancouver, BC). The tumor cell fraction was incubated (20min) in Permeabilization Reagent (100μL) and Nucleic Acid Dye (50μL; Janssen Diagnostics). After washing, cells bound by PE-conjugated antibodies (HLA/EpCAM) were immunomagnetically labelled using the EasySep PE Positive Selection kit (StemCell Technologies) and transferred to a MagNest™ for analysis using the CSS. EpCAM/HLA^+^/DAPI^+^/CD45^−^ cells with a round/oval morphology were classified as CTCs.

### *In vivo* CTC and metastasis assays

Animal experiments were conducted under protocol# 2012-031 approved by Western University's Animal Care Committee. PCa cells were prepared in sterile Hank's buffered saline (Life Technologies) and injected (1×10^6^ cells/40μL per mouse) orthotopically into 6-8wk old male athymic nude (*nu/nu*) mice (Harlan Sprague-Dawley) via the right dorsolateral lobe of the prostate as described [53, 54]. Briefly, a low midline abdominal incision of approximately 3-4 mm was made in anesthetized mice. The bladder was gently lifted and the right dorsolateral lobe was identified. The cell solution was slowly injected into the prostate gland before replacing the bladder and suturing the muscle and skin layers closed. At 2,4,8,12 and 16wks post-injection, mice were sacrificed, necropsies performed, and tissues collected. Prostates and bladders were weighed as surrogates of primary tumor volume. Blood (100μL) was collected and processed using both CTC assays (50μL/assay) to assess differences in CTC dissemination and kinetics. Whenever possible, CTC sub-cell lines were generated using excess blood lysed (NH_4_Cl) and cultured.

Throughout the study mice were occasionally sacrificed at modified timepoints (±1-2wks) due to morbidity and/or technical issues. To facilitate statistical analysis, mice were categorized based on time of sacrifice (1-3, 4-6, 8-10, 11-13 or 14-16wks). For simplicity, data is presented at the initially defined timepoints (2, 4, 8, 12, 16wks). Due to unexpectedly rapid progression of the PC-3M cell line, CTCs in this group could only be assessed at 2-4wks.

### Histology and immunohistochemistry

At necropsy, tissues were formalin-fixed, paraffin-embedded, sectioned (4μm) and stained (hematoxylin and eosin [H&E]). Serial sections were deparaffinized (xylene) and rehydrated (graded series of alcohols [100/95/80/75%]) prior to staining. Antigen retrieval was performed (10mM sodium citrate buffer/0.05% Tween-20 [100°C; 20min]) before incubation with BLOXALL Endogenous Peroxidase and Alkaline Phosphatase Blocking Solution (Vector Laboratories). Staining for E-cadherin (1:100) and N-cadherin (1:750) was performed (Polink DS-MR-Hu kit [GBI Labs, Bothell, WA]). Slides were imaged (40x) using an Aperio ScanScope (Aperio Technologies, Vista CA).

### Statistical analysis

Statistical analysis was performed using GraphPad Prism 5.0 (San Diego, CA) and/or SPSS (IBM, Armonk, NY) as detailed in Figure Legends, with p≤0.05 considered statistically significant.

## SUPPLEMENTARY FIGURES AND TABLES


