# Supplementary material for: Epithelial-to-mesenchymal transition leads to disease-stage differences in circulating tumor cell detection and metastasis in pre-clinical models of prostate cancer
Source: Oncotarget. 2016 Oct 15;7(46):76125–39. doi: 10.18632/oncotarget.12682 (PMC5342801; doi:10.18632/oncotarget.12682)
Supplement: Supplementary file 1 [file oncotarget-07-76125-s001.pdf]

Epithelial-to-mesenchymal transition leads to disease-stage differences in circulating tumor cell detection and metastasis in pre-clinical models of prostate cancer

SUPPLEMENTARY FIGURES AND TABLES

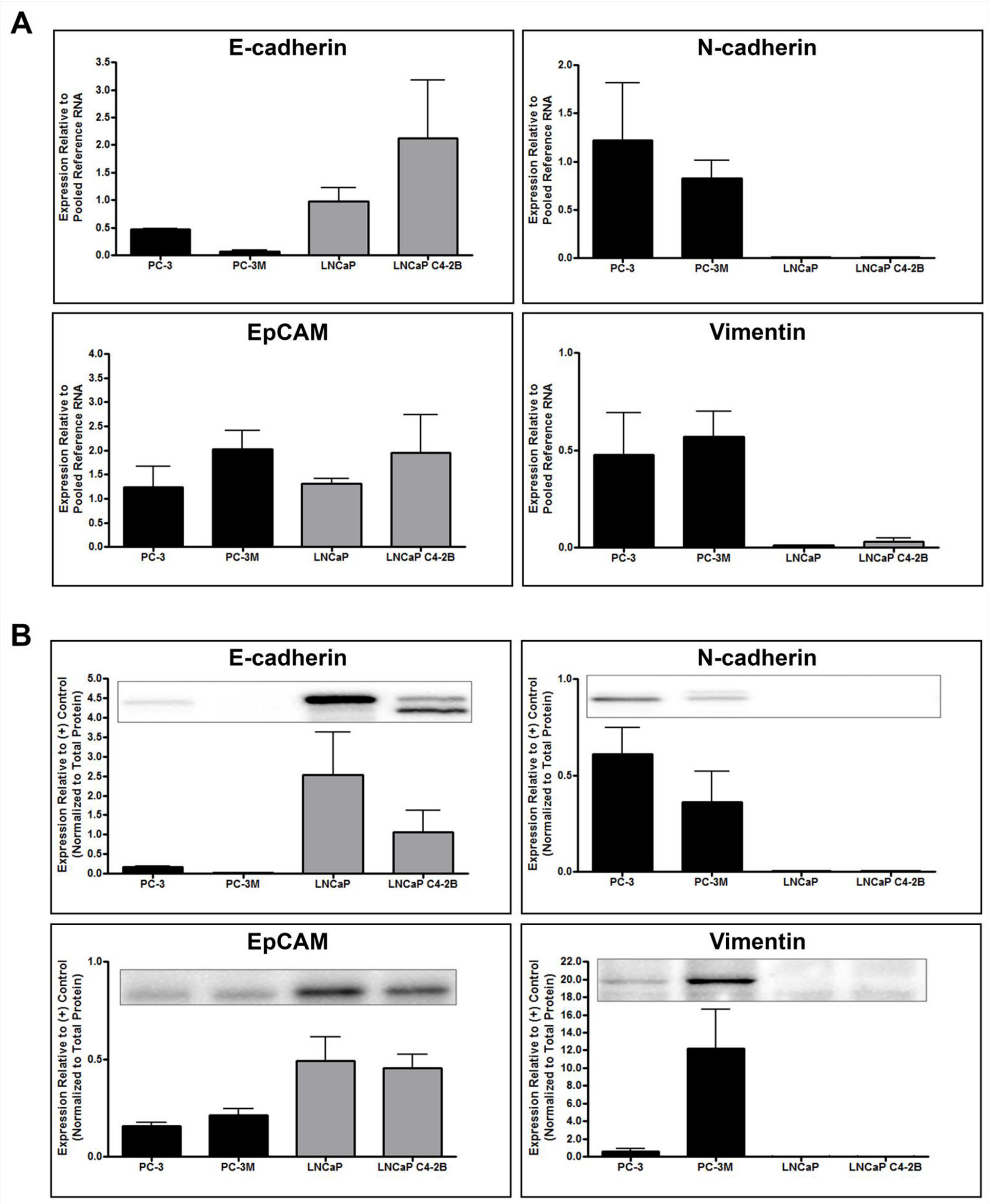

Supplementary Figure S1: Human prostate cancer cell lines display differences in epithelial-to-mesenchymal transition (EMT) phenotype at the RNA and protein level. A-B. The expression of epithelial-associated markers E-cadherin and EpCAM and mesenchymal-associated markers N-cadherin and vimentin is associated with previously reported cell aggressiveness and *in vivo* metastatic capacity of the human prostate cancer cell lines PC-3, PC-3M (*black bars*), LNCaP and LNCaP C4-2B (*grey bars*). (A) RT-qPCR analysis of mRNA expression; data presented as relative expression (mean ± SEM) compared to pooled reference RNA (n=3). (B) Immunoblot analysis of protein expression; presented as quantitative densitometric data (mean ± SEM) relative to appropriate positive control cell lines and normalized to total protein loaded as assessed by amido black staining, and as representative immunoblots shown as cropped gel images (n=3).

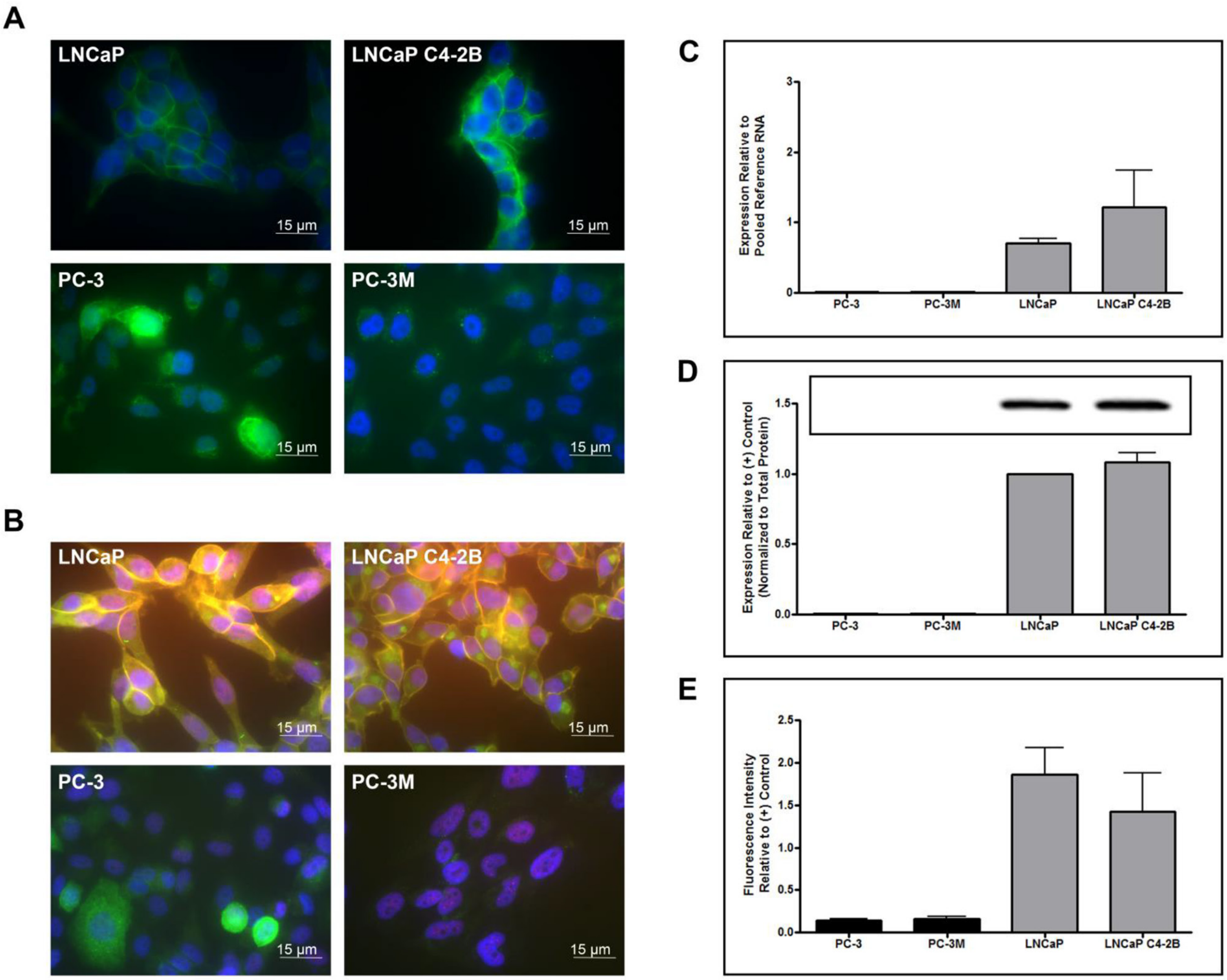

Supplementary Figure S2: E-cadherin cell membrane localization is aberrant in human prostate cancer cell lines that do not express α-catenin. A. Representative immunofluorescent images of E-cadherin (*green*) and DAPI (*blue*) stained PC-3, PC-3M, LNCaP, and LNCaP C4-2B cell lines cultured on glass chamber slides. **B.** Representative immunofluorescent images of co-localization (*yellow*) of E-cadherin (*green*) and α-catenin (*red*) in PC-3, PC-3M, LNCaP, and LNCaP C4-2B cell lines cultured on glass chamber slides (DAPI [blue]). Images were obtained at 60x magnification, scale bars = 15 µm (n=3). **C.** RT-qPCR analysis of mRNA; data presented as relative expression (mean ± SEM) compared to pooled reference RNA (n=3). **D.** Immunoblot analysis of protein expression; presented as quantitative densitometric data (mean ± SEM) relative to an appropriate positive control cell line and as a representative immunoblot, shown as a cropped gel image (n=3). **E.** Flow cytometry analysis; presented as relative fluorescence intensity (expression; mean ± SEM) compared to an appropriate positive control cell line (n=3).

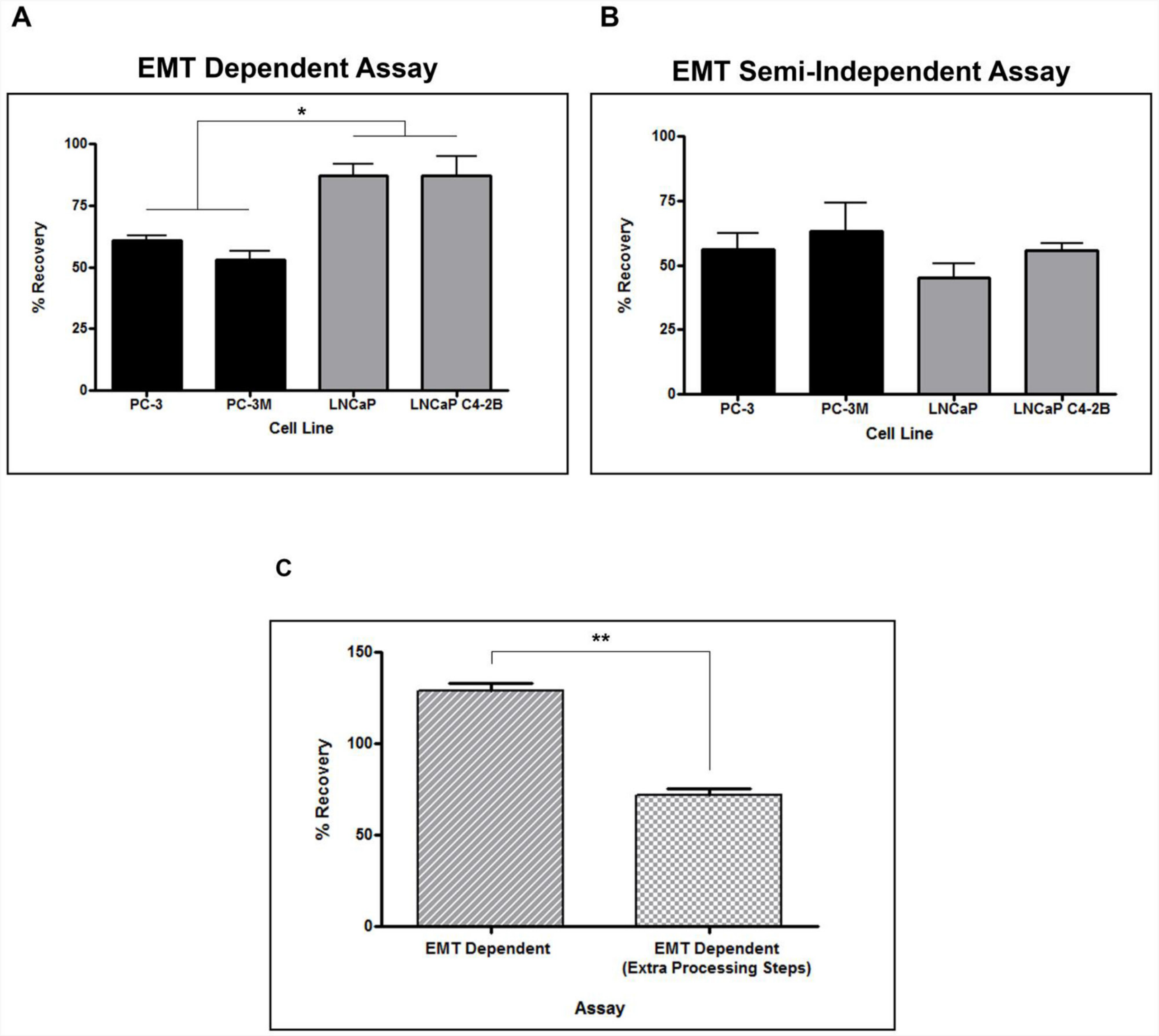

Supplementary Figure S3: CTC recovery using the CellSearch® system is reduced in human prostate cancer cells with a mesenchymal phenotype. Prostate cancer cells were counted by hemocytometer and spiked at a concentration of 1,000 tumor cells/50µl of whole mouse blood. 50µl of mouse blood was subsequently processed using 1 of 2 mouse-adapted protocols and CTC recovery was measured as a percentage recovery of the number of spiked cells. **A.** CTC recovery using the EMT-dependent assay is significantly lower in cells with a more mesenchymal and metastatic phenotype. **B.** CTC recovery using the EMT semi-independent assay is lower relative to the EMT dependent assay. However, equivalent recovery is observed across all 4 cell lines regardless of EMT phenotype. **C.** Reduced recovery observed when using the EMT semi-independent assay was further investigated and determined to be as a result of extra processing steps required in this protocol. Data are presented as the mean ± SEM (n=3). Analysis of differences in CTC recovery using the 2 assays was performed using 1-way ANOVA with Tukey's post-test for multiple comparisons. * = significantly different ($p \leq 0.05$).

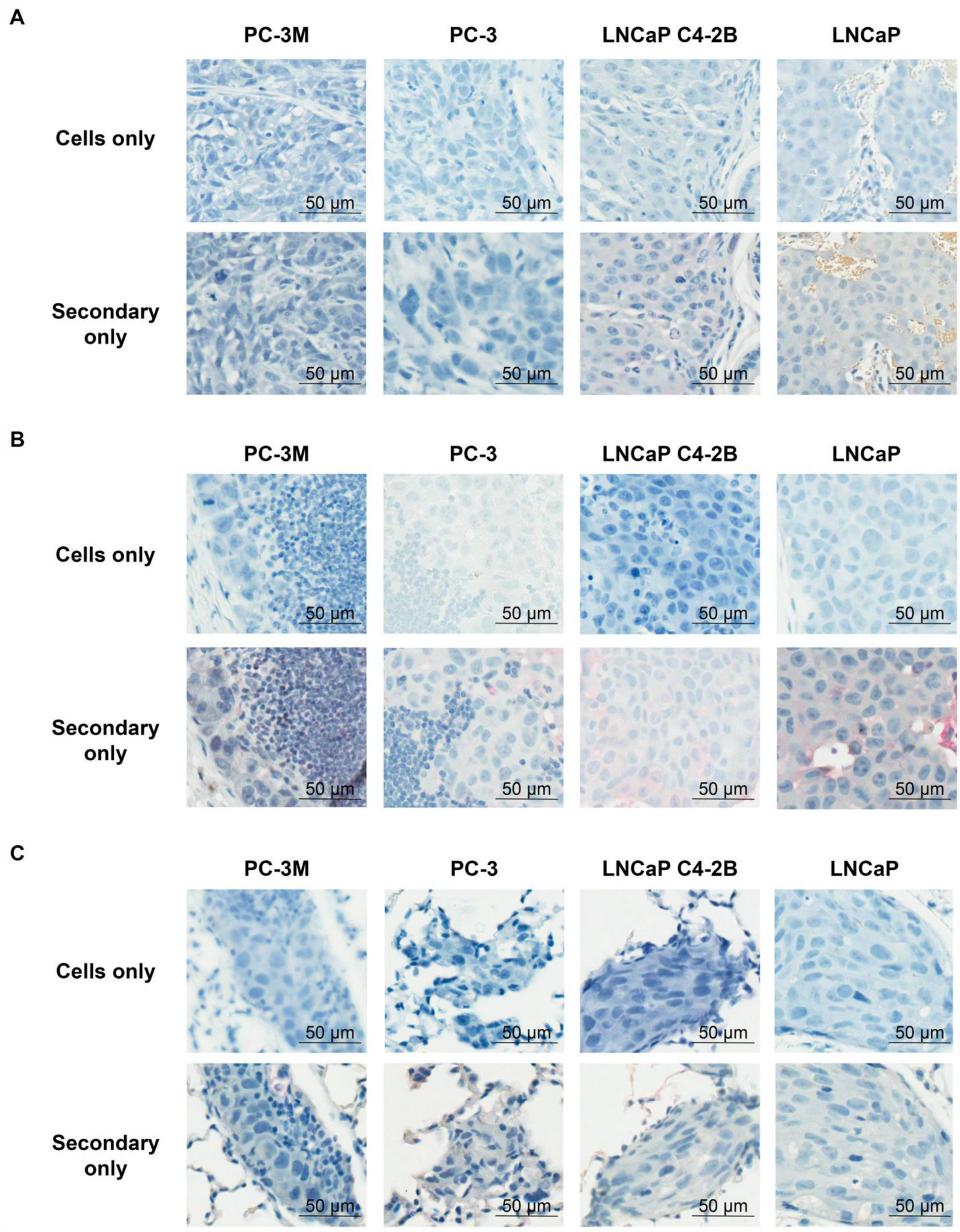

Supplementary Figure S4: Cells only and secondary antibody controls for IHC. Cells only and secondary only controls for IHC performed on **A.** primary tumors, **B.** lymph node metastases, and **C.** lung metastases. Histological sections are presented at 40x magnification. Scale bars=50 μm.

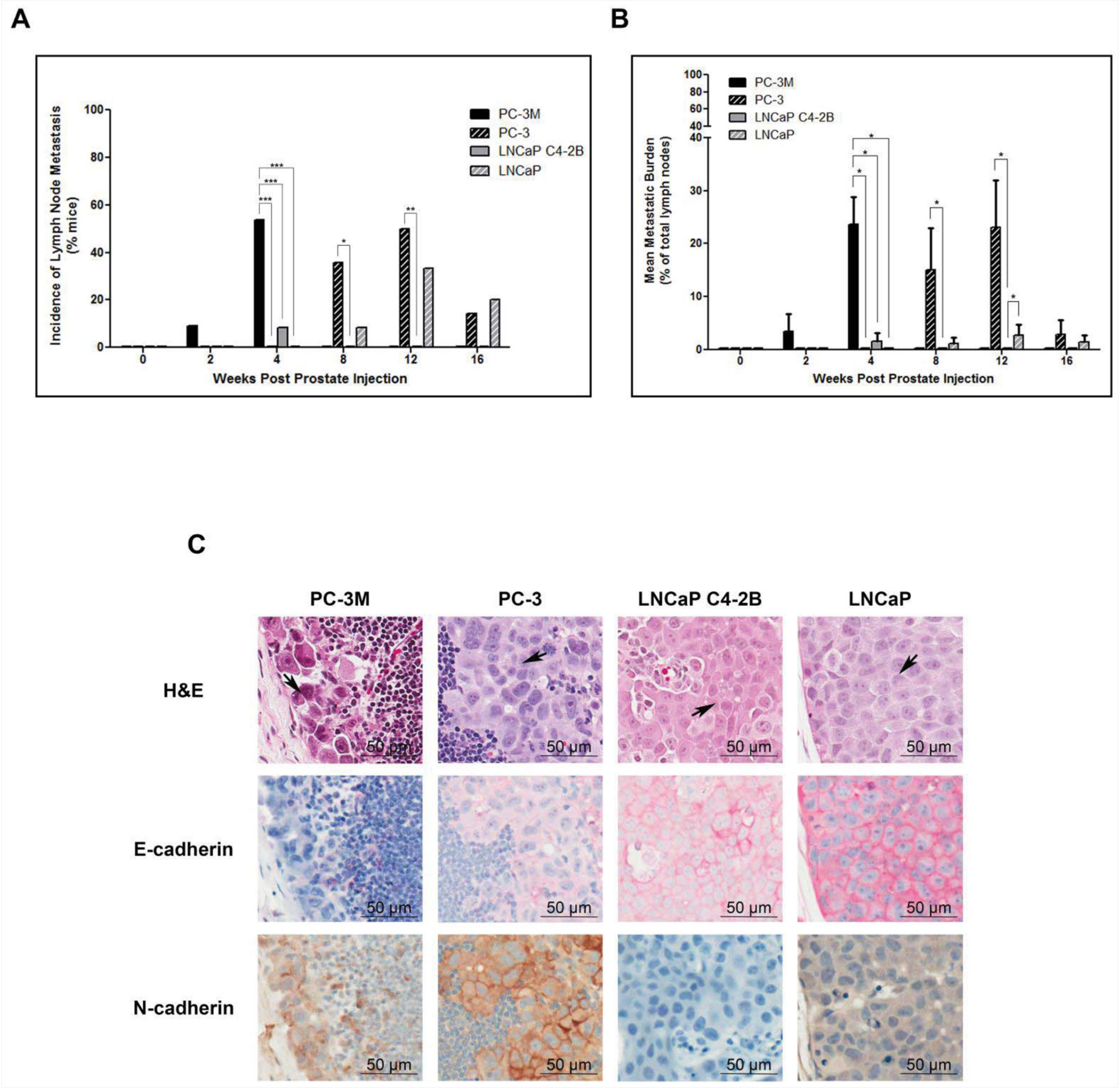

Supplementary Figure S5: The mesenchymal PC-3M and PC-3 cell lines exhibit a greater incidence of lymph node metastases and mean lymph node metastatic burden. A. Incidence of lymph node metastasis in mice injected with PC-3M, PC-3, LNCaP C4-2B, and LNCaP prostate cancer cell lines based on microscopic histological examination of formalin fixed, H&E stained tissue following orthotopic injection. Data are presented as the percentage of mice per cell line per timepoint with detectable lymph node metastases (n=7-39 mice/group). **B.** Quantitative analysis of tumor burden (mean % of lymph nodes occupied by tumor) following orthotopic injection of prostate cancer cell lines. Data are presented as the mean ± SEM (n=7-39 mice/group). Differences in the incidence of lymph node metastasis were assessed using Fisher's Exact Test. Differences in metastatic burden to the lymph nodes were assessed by Wilcoxon Score followed by a Kruskal-Wallis test. * = significantly different ($p \leq 0.05$). **C.** Representative H&E and IHC (E-cadherin and N-cadherin) images of lymph node metastases for each investigated cell line. Histological sections are presented at 40x magnification. Arrowheads on H&E images indicate regions of tumor within the given tissue. Scale bars=50 μm.

Supplementary Table S1: Forward and reverse primers used for qPCR analysis

Target Gene	Forward Primer (5' → 3')	Reverse Primer (5' → 3')
E-cadherin	TGCTGATGCCCCCAATACCCCA	GTGATTTCCTGGCCCACGCCAA
N-cadherin	TGACTCCAACGGGGACTGCACA	AGCTCAAGGACCCAGCAGTGGA
EpCAM	CGACTTTTGCCGCAGCTCAGGA	GGGCCCCTTCAGGTTTTGCTCT
Vimentin	AACCAACGACAAAGCCCGCGTC	TTCCGGTTGGCAGCCTCAGAGA
α-catenin	CCACGTTTTACTGAGCAAGT	AGTCAGAGTCATCCAACTCC
GAPDH	TCCATGGCACCGTCAAGGCTGA	GCCAGCATCGCCCCACTTGATT

Sequence specific primers were designed based on gene sequence information from the National Center for Biotechnology Information (NCBI; Bethesda, MD).

Supplementary Table S2: Anti-human antibodies used for immunoblot analysis

Target Protein	Clone	Commercial Source	1° Ab Host	kDa	1° Ab Conditions
N-Cadherin	EPR1791-4	Abcam	Rabbit	100	1:1,000 (1 hr @ RT)
E-Cadherin	36/E-cadherin	BD Biosciences	Mouse	120	1:20,000 (1 hr @ RT)
Vimentin	V9	Millipore	Mouse	60	1:1,000 (1 hr @ RT)
EpCAM	E144	Abcam	Rabbit	39	1:1,000 (1 hr @ RT)
α-catenin	EP1793Y	Abcam	Rabbit	100	1:50,000 (1 hr @ RT)
β-Actin	Polyclonal	Sigma	Rabbit	42	1:5,000 (1 hr @ RT)

Supplementary Table S3: Anti-human antibodies used for flow cytometry analysis

Target Protein	Clone	Commercial Source	1° Ab Amount per 5 × 10^5 cells
N-Cadherin	EPR1791-4	Abcam	0.40 μg
E-Cadherin	36/E-cadherin	BD Biosciences	0.80 μg
Vimentin	V9	Millipore	0.80 μg
EpCAM	E144	Abcam	0.15 μg
α-catenin	EP1793Y	Abcam	0.35 μg
CK 8/18/19	Polyclonal	Janssen Diagnostics (CellSearch)	100 μl
